# Supplementary material for: Content-rich biological network constructed by mining PubMed abstracts
Source: BMC Bioinformatics. 2004 Oct 8;5:147. doi: 10.1186/1471-2105-5-147 (PMC528731; doi:10.1186/1471-2105-5-147)
Supplement: Additional File 5 — The original Chilibot query results of the term "long-term potentiation (LTP)" and 22 other terms, limiting the latest references analyzed to the years 1990, 1995, 2000, and 2004. [file 1471-2105-5-147-S5.bz2 › chilibotAdditionalFile5/ltp1995/html/NMDA_CAMKII.html]

 


 **NMDA** and **CAMKII** 
  
Found 14 abstracts in PubMed,  **14 abstracts were retrieved and analyzed**.  


---

 Search Google  |
 PDF files only 
|  EDU domain only 

---

**Interactive relationship** (e.g. stimulation, inhibition, etc)

- The biphasic regulation of  **CaMKII**  alpha mRNA may be of considerable functional importance for the long term response of granule cells to local stimulation of  **NMDA**  receptors or NO release.  Ref: 7476022 Brain Res Mol Brain Res, 1995
- Here we report that in acutely isolated rat spinal dorsal horn DH neurons, the alpha amino 3 hydroxy 5 methyl 4 isoxazole propionic acid AMPA kainate and N methyl D aspartate  **NMDA**  receptors can be regulated by endogenous and exogenous calcium calmodulin dependent protein kinase II  [ **CAMKII** ]  CaM KII.  Ref: 7884477 J Neurophysiol, 1994

**Parallel relationship** (e.g. studied together, co-existance, homology, etc.)

- We report here that at short time periods 1 6 h after injections of  **NMDA**  or sin 1 molsidomine, there is an increase in the levels of the mRNA encoding the alpha subunit of calcium calmodulin dependent protein kinase II  **CaMKII**  alpha, consistent with a report of elevated  **CaMKII**  alpha mRNA in postsynaptic neurons in the CA1 region of the hippocampus following LTP induction 54.  Ref: 7476022 Brain Res Mol Brain Res, 1995
- However, we also report that 24 h after injection of  **NMDA**  or sin 1, there is a dramatic decrease in  **CaMKII**  alpha mRNA levels in the vicinity of the injection.  Ref: 7476022 Brain Res Mol Brain Res, 1995
- A specific inhibitor of calcium calmodulin dependent protein kinase II  [ **CAMKII** ]  provides neuroprotection against  **NMDA**  and hypoxia hypoglycemia induced cell death.  Ref: 7538570 J Neurosci, 1995
